# Effects of *Sargassum thunbergii* Extract on Skin Whitening and Anti-Wrinkling through Inhibition of *TRP-1* and *MMPs*

**DOI:** 10.3390/molecules26237381

**Published:** 2021-12-05

**Authors:** Da-Hye Gam, Jae-Hyun Park, Ji-Woo Hong, Seong-Jin Jeon, Jun-Hee Kim, Jin-Woo Kim

**Affiliations:** 1 Department of Food Science, Sun Moon University, Natural Science 118, 70 Sunmoon-ro 221, Tangjeong-myeon, Asan-si 336-708, Chungnam, Korea; ank7895@naver.com (D.-H.G.); gengihoo@naver.com (J.-H.P.); hgw130@naver.com (J.-W.H.); zzx01010@naver.com (S.-J.J.); jun981014@naver.com (J.-H.K.); 2 FlexPro Biotechnology, Natural Science 128, 70 Sunmoon-ro 221, Tangjeong-myeon, Asan-si 336-708, Chungnam, Korea

**Keywords:** *S. thunbergii*, optimization, skin-whitening, anti-wrinkling, *TRP-1*, *MMP-1*, *MMP-9*

## Abstract

*Sargassum thunbergii* has been traditionally used as an edible and medicinal material in oriental countries. However, the skin-whitening and anti-wrinkling effects of *S. thunbergii* have not yet been investigated. This study was conducted to establish optimal extraction conditions for the production of bioactive compounds with antioxidant activity as well as skin-whitening and anti-wrinkle effects using ultrasound-assisted extraction (UAE) in *S. thunbergii.* The extraction time (5.30~18.7 min), extraction temperature (22.4~79.6 °C), and ethanol concentration (0.0~99.5%), which are the main variables of the UAE, were optimized using a central composite design. Quadratic regression equations were derived based on experimental data and showed a high coefficient of determination (R^2^ > 0.85), demonstrating suitability for prediction. The optimal UAE condition for maximizing all dependent variables, including radical scavenging activity (RSA), tyrosinase inhibitory activity (TIA), and collagenase inhibitory activity (CIA), was identified as an extraction time of 12.0 min, an extraction temperature of 65.2 °C, and ethanol of 53.5%. Under these conditions, the RSA, TIA, and CIA of *S. thunbergii* extract were 86.5%, 88.3%, and 91.4%, respectively. We also confirmed *S. thunbergii* extract had inhibitory effects on the mRNA expression of tyrosinase-related protein-1, matrix metalloproteinase-1, and matrix metalloproteinase-9, which are the main genes of melanin synthesis and collagen hydrolysis. Liquid chromatography-tandem mass spectrometry was used to identify the main phenolic compounds in *S. thunbergii* extract, and caffeic acid was identified as a major peak, demonstrating that high value-added ingredients with skin-whitening and anti-wrinkling effects can be produced from *S. thunbergii* and used for developing cosmetic materials.

## 1. Introduction

Melanogenesis is a physiological process that leads to the synthesis of melanin pigments [1]. Melanin is a black or brown pigment secreted from the melanocytes present in the basal layer of the epidermis and determines the skin, eyes, and hair color [2]. However, excessive generation of melanin pigments can lead to hyperpigmentation-related diseases, such as malignant melanomas [3]. Tyrosinase, the main enzyme in the melanin biosynthesis pathway, promotes the hydroxylation of L-tyrosine to L-DOPA (L-3,4-dihydroxyphenylalanine), and then promotes the oxidation of L-DOPA to dopachrome and dopaquinone, which synthesizes melanin through the auto-oxidation process by tyrosinase-related proteins (TRP-1 and 2) through several stages [4,5,6]. To date, methods of inhibiting melanin formation by impeding tyrosinase-related proteins have been widely used in the cosmetics industry for the development of skin-whitening agents [7,8]. However, kojic acid, azelaic acid, and hydroquinone, conventionally used skin-whitening ingredients, have been reported to induce allergies as well as cause skin toxicity and cancer [9]. Therefore, producing a safer, more effective skin-whitening agent that is based on natural ingredients is considered imperative [10].

Collagen is an extracellular matrix (ECM) protein that protects the skin by giving it strength and tension; it also helps delay the aging process by preventing wrinkles and moisture loss. ECM can be decomposed by matrix metalloproteinases (MMPs) [11]. MMP-1, commonly known as collagenase, partially decomposes the type 1 collagen that makes up the skin, whereas MMP-9, known as gelatinase, additionally depolymerizes the collagen hydrolyzed by MMP-1 [12]. In addition, It has been reported that oxidative stress induced by reactive oxygen species (ROS) accelerates the synthesis of these enzymes, leading to the degradation of ECM and, ultimately, wrinkle formation [13]. Therefore, it is necessary to find natural ingredients that can inhibit the expression of TRP and MMPs and that contain antioxidants that can remove reactive oxygen species to prevent skin aging by reducing pigmentation and wrinkling of the skin [14]. Recently, as the functional ingredients for cosmetics have been developed mainly in land plants, limitations have begun to arise in exploring new species and a stable supply of natural ingredients [15]. Consequently, interest in and demand for natural ingredients derived from marine plants has increased, and a variety of new ingredients have been identified from marine resources [16].

*Sargassum thunbergii* is a species of brown macroalgae belonging to the gulfweed family and is native to the coast of Korea and China [17]. It is recognized as a marine pollutant that causes damage to seaweed and fish farms by depleting dissolved oxygen [18]. A few of them are used as anthelmintic drugs in traditional therapy or as compost [19]. However, an anticancer ingredient was identified from its extract in 1995; since then, it has garnered attention as a macroalgae with a high potential for use in the manufacture of novel bioactive compounds [20]. Extraction of bioactive compounds using conventional processes, including mechanical expelling, supercritical extraction, microwave extraction, and ultra-high-pressure extraction, are associated with limitations such as the need to use excess solvent, low extraction yield, and high energy consumption [21]. Developing new extraction methods is one of the major challenges in technological innovation to secure bioactive compounds from macroalgae [22]. Among the conventional extraction processes, ultrasound-assisted extraction (UAE) is particularly attractive due to its simplicity, low equipment cost, high extraction yield from different matrices, low energy consumption, a lower amount of solvent required, and less time [23]. UAE is known to involve high-frequency sound waves of 20–100 kHz [24]. The extraction yield is enhanced using ultrasound, and this is attributed to the disruption of the plant tissues, reduction in particle size, and increased mass transfer of extracts to the solvent caused by the collapse of the bubbles that are produced by repeated acoustic cavitation [25,26]. Due to these advantages, UAE is recognized as an inexpensive, renewable, and efficient process that is widely used in the food industry to extract functional ingredients from terrestrial and aquatic biomass [27].

Thus, in this study, we applied UAE to extract bioactive compounds from *S. thunbergii* and derived the optimal UAE conditions that allow the maximum extraction of antioxidants as well as skin-whitening and anti-wrinkling ingredients using statistically based optimization, and various The inhibitory effects of *TRP-1*, *MMP-1*, and *MMP-9* gene expression by *S. thunbergii* extract were evaluated to verify the skin-whitening and anti-wrinkling effects of the derived bioactive compounds and confirm the possibility of utilizing *S. thunbergii* extract as a functional cosmetic ingredient.

## 2. Results and Discussion

### 2.1. Design of the Experiment

Fitting the model is crucial to interpret the accuracy of the response surface methodology (RSM) mathematical model for predicting the radical scavenging activity (RSA), tyrosinase inhibitory activity (TIA), and collagenase inhibitory activity (CIA) of *S. thunbergii* extract. The central composite design (CCD) of RSM is an experimental design method that statistically analyzes the response surface produced independently or by the interaction of two independent variables affecting the responses. The CCD has the advantage of effectively estimating curvature using the center point and multiple axial points to predict the optimal conditions [28,29,30]. In this study, CCD was applied to predict the optimal UAE conditions to maximize the responses, including the RSA, TIA, and CIA, of *S. thunbergii* extract. The 5 levels (−α, −1, 0, 1, α) were coded, and 17 experimental runs were performed as a base on CCD (Table 1). Based on our previous studies, 3 key independent variables, including extraction time (5.30~18.7 min), extraction temperature (22.4~79.6 °C), and ethanol concentration (0~99.5%), were selected to obtain the maximum level of dependent variables [31]. In developing the quadratic regression model, the experimental variables were coded according to the following equation.

(1) $$x_{i}=\left(X_{i}-X_{0}\right)/\Delta X$$

where *x_i_* is the coded value of the variable *X_i_*; *X*_0_ is the value of *X* at the center point; and Δ*X* is the step change value.

Experimental values for 17 conditions with differences in extraction time, extraction temperature, and ethanol concentration are shown in Table 2.

### 2.2. Effects of UAE Conditions on RSA

According to the 17 conditions applied to the extraction of *S. thunbergii* using UAE, RSA was 2.37~89.9%, with the maximum value at 12.0 min extraction time, 51.0 °C extraction temperature, and 50.0% ethanol concentration and the minimum value at 12.0 min extraction time, 51.0 °C extraction temperature, and 99.5% ethanol concentration; this indicates that the ethanol concentration had the greatest effect on RSA (Table 2). As suggested by the Design-Expert software, a quadratic regression equation was selected and fitted for all three independent variables and responses. In terms of coded values, the predicted responses for the RSA, TIA, and CIA could be expressed using quadratic regression equations via multiple regression analysis (Table 3). The CCD model coefficients were validated using analysis of variance (ANOVA) for the response variables of the quadratic regression models summarized in Table 4. If the coefficient of determination (R^2^), which represents the agreement between the experimental and predicted values, is close to 1, it implies acceptable goodness of fit [32]. The R^2^ of the quadratic regression equation for predicting the optimal UAE condition with RSA was 0.8554, establishing that ≥85.5% of the resulting predicted value can be completely explained, thus recognizing the suitability of the quadratic regression equation (Table 3).

The significance of each model variable was determined using *p* values; a *p* value of <0.05 indicates significance whereas a *p* value of >0.05 indicates insignificance on the RSA [33]. The ANOVA results of the optimization study indicated that the model was significant (*p* = 0.0283), which was less than the set significance level, indicating that significance was recognized within 5%. Therefore, the results indicate that the models could efficiently predict the RSA, TIA, and CIA of *S. thunbergii* extract when independent variables were within the ranges depicted here. Upon checking the significance of each independent variable, we found the ethanol concentration had the most effect on RSA (*p* = 0.0025), whereas the effects of extraction time (*p* = 0.7418) and temperature (*p* = 0.1622) were insignificant (Table 4).

**Figure 1:**
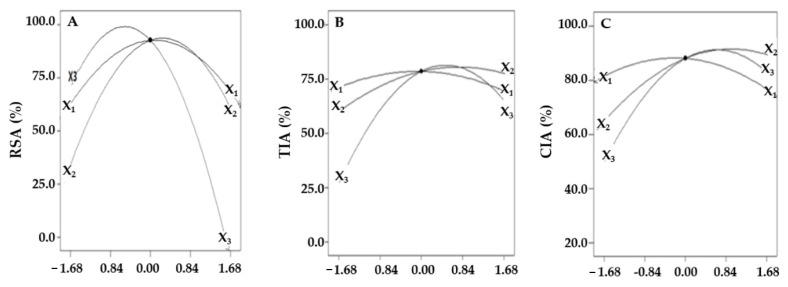
Perturbation plots showing the effects of each of the independent variables on RSA (**A**), TIA (**B**), and CIA (**C**) while fixing other variables at center points. X_1_ = extraction time (min); X_2_ = extraction temperature (°C); X_3_ = ethanol concentration (%).

To evaluate the effect of each independent variable on the dependent variable, we expressed the change in RSA according to the extraction time, extraction temperature, and ethanol concentration as a perturbation plot (Figure 1A). 

**Figure 2:**
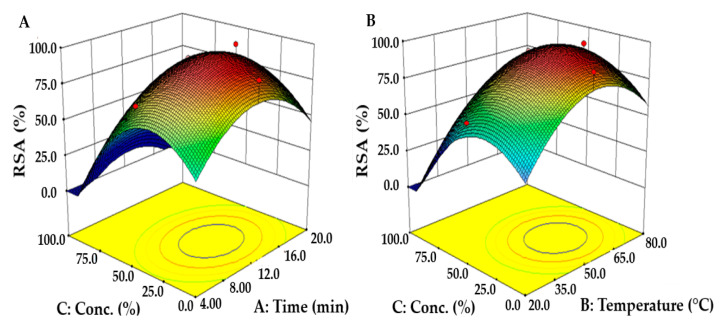
Response surface plots showing the interactive effects of (**A**) extraction time and ethanol concentration and (**B**) extraction temperature and ethanol concentration on the RSA of *S. thunbergii* extract. The third variable was fixed at the central point of the CCD.

Consequently, the highest value appeared at 14.1 min of extraction time and then decreased; the maximum value was confirmed at 60.7 °C and 46.2% for the extraction temperature and ethanol concentration, respectively. However, when visualizing the rate of change in RSA due to the interactions between variables using the three-dimensional response surface curve, change in extraction time and temperature had little effect on RSA, whereas the ethanol concentration had a significant effect (Figure 2A,B). 

This was similar to the results of the study by Kim et al. identifying the effect of solvent concentration on the RSA of *Gynostemma pentaphyllum* extract [34]. RSA tended to increase to a maximum and then decreased with the ethanol concentration, showing the maximum value at 48.1% of ethanol concentration. The polarity change of the extraction solution due to the mixing of distilled water and ethanol leads to an increase in the antioxidant effect of *G. pentaphyllum* and *S. thunbergii* extracts. In addition, this is consistent with the results reporting that water-soluble bioactive compounds produced by hot-water extraction from algae show less antioxidant activity and that extracts that use 50% ethanol show higher antioxidant activity, suggesting that the use of a binary solvent (water and ethanol) in producing bioactive compounds is beneficial in increasing the extraction yield [35,36].

### 2.3. Effects of UAE Conditions on TIA

The TIA of *S. thunbergii* extracts according to 17 UAE conditions applied to the experiment is shown in Table 2. The maximum TIA value of 92.6% was identified at 12.0 min, 79.6 °C, and 50.0% and the minimum value of 55.3% was predicted at 12.0 min, 51.0 °C, and 0.0% of extraction time, extraction temperature, and ethanol concentration, respectively. Consequently, extraction temperature and ethanol concentration were confirmed to have a significant effect on TIA. On the basis of the experimental results, we derived a quadratic regression equation using CCD and used it to predict the optimal UAE conditions (Table 3). The R^2^ was 0.8591, indicating an 85.91% match between the values of the predicted model and experimental data and implying that the quadratic regression equation was suitable for TIA prediction. For the responses of RSA, TIA, and CIA, the models were highly significant when the computed F-values were greater than the tabulated F-value and the probability values were low (*p* < 0.001); this indicates that the individual terms in each response model were significant in terms of the interaction effect [37]. ANOVA was applied to statistically evaluate the significant effect of the quadratic regression equation. The experimental model was significant (*p* = 0.0262), indicating a level of significance within 5% (Table 4).

**Figure 3:**
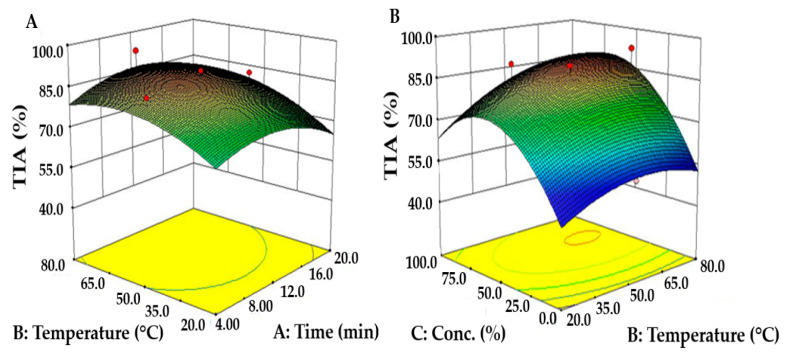
Response surface plots showing the interactive effects of (**A**) extraction time and ethanol concentration, (**B**) extraction temperature and ethanol concentration on TIA of *S. thunbergii* extract. The third variable was fixed at the central point of the CCD.

When we visualized the rate of TIA change with the change of a single variable when fixing the values of other variables, TIA variation due to ethanol concentration was the largest, with a maximum TIA found at 76.8% ethanol concentration (Figure 1B). The interactions of independent variables are visualized using the three-dimensional response surface curve by simultaneously changing two variables (Figure 3). 

As the extraction temperature and time increase, TIA increases initially; however, the variation range is not large, so we reconfirmed that the interactive effect of extraction temperature and time is not significant, as determined using ANOVA (Figure 3A). Conversely, TIA increased and decreased again with ethanol concentration, with the maximum TIA predicted to be at 75.6% ethanol concentration (Figure 3B). This result is consistent with those of the study by Park et al., which showed that 70~80% ethanol concentration leads to a higher TIA than water in the extraction of bioactive compounds from wild rice extract [38]. That study reported that ethanol concentration was a major variable in TIA and tends to vary in proportion with the ethanol concentration.

### 2.4. Effects of UAE Conditions on CIA

When we measured the CIA under each of 17 conditions, we found that the maximum CIA value was 92.3% at 16.0 min, 63.0 °C, and 80.0% and the minimum CIA was 48.1% at 12.0 min, 51.0 °C, and 0.0% of extraction time, extraction temperature, and ethanol concentration, respectively (Table 2). The quadratic regression equation generated according to extraction time, temperature, and ethanol concentration had an R^2^ of 0.9237, implying that the sample variation of 92.37% was attributed to the independent variables, and only 7.63% of the total variations could not be explained by the model (Table 3). This indicates a good degree of correlation between the predicted and experimental values of the CIA and recognizes its suitability in predicting the experimental model [39]. ANOVA demonstrated statistical significance (*p* = 0.0037) below a significance level of 1% and confirmed that the extraction temperature (*p* = 0.0030) and ethanol concentration (*p* = 0.0006) among the linear terms were independent variables that significantly affected CIA (Table 4).

**Figure 4:**
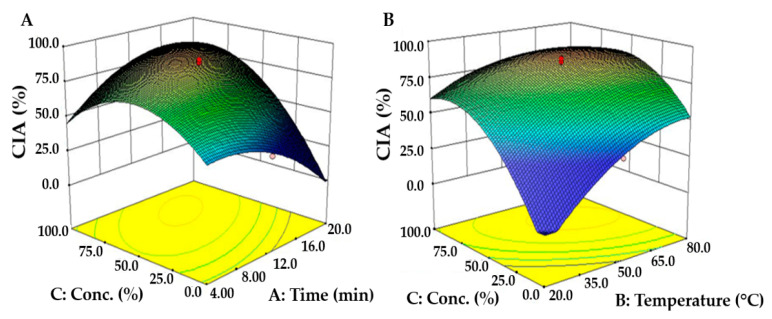
Response surface plots showing the interactive effects of (**A**) extraction time and ethanol concentration, (**B**) extraction temperature and ethanol concentration on CIA of *S. thunbergii* extract. The third variable was fixed at the central point of the CCD.

To evaluate the effects of each independent variable on CIA, we compared CIA with the change in one variable using a perturbation plot (Figure 1C). As the independent variable increased, CIA initially increased to the maximum value, and the ethanol concentration was found to be the most influential. The three-dimensional response surface curve represented CIA change due to the interactive effects of independent variables, which tended to increase and decrease with extraction time and ethanol concentration. CIA increased with increasing extraction time and ethanol concentration, showing the maximum CIA at 12.1 min of extraction time and 73.6% ethanol concentration. The changes in CIA with extraction temperature and ethanol concentration at a constant extraction time also tended to be the same; however, the variations in CIA with ethanol concentration were confirmed to be more significant (Figure 4B). 

The maximum value of CIA predicted by CCD was 93.8% with an extraction time of 14.5 min, extraction temperature of 65.1 °C, and an ethanol concentration of 69.3%. This was more than twice as high as the 39.4% and 40.3% of CIA for the green tea and white tea hot-water extracts found in a previous study [40]. In conclusion, the *S. thunbergii* extract was considered capable of being utilized as a functional cosmetic ingredient to reduce wrinkles, as it restrains the activity of collagenase.

### 2.5. Optimization of the UAE Process

**Figure 5:**
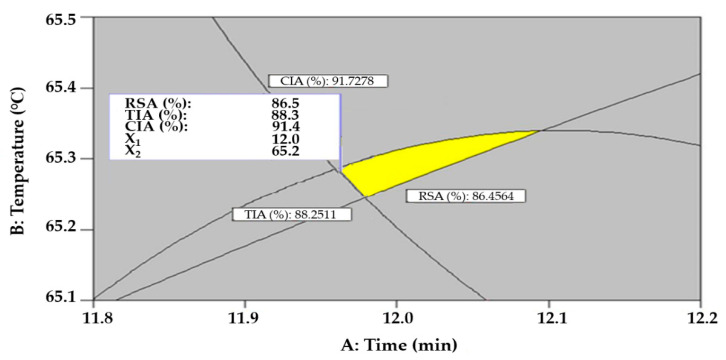
Superimposing contour map for the simultaneous maximization of RSA, TAI, and CAI to derive conditions that can maximize antioxidant, skin-whitening, and anti-wrinkle effects. Ethanol concentration was fixed at the optimum level of 53.5%.

To identify the optimal UAE condition for the extraction of skin-whitening and anti-wrinkle bioactive compounds from *S. thunbergii* extract, we obtained an optimal point for maximizing the dependent variables by overlapping the individual response surfaces of RSA, TIA, and CIA (Figure 5). 

When the range of independent variables was limited to an extraction time of 5.30~18.7 min, extraction temperature of 22.4~79.6 °C, and ethanol concentration of 0~99.5%, the optimal UAE condition was predicted to be 12.0 min extraction time, 65.2 °C extraction temperature, and 53.5% ethanol concentration. The optimal UAE condition was derived based on the criteria of minimizing extraction time because a short process time is beneficial in reducing process costs. Under the optimal UAE condition derived, 86.5%, 88.3%, and 91.4% of RSA, TIA, and CIA, respectively, were predicted. In previous studies, Yuan et al. reported that the optimal conditions for bioactive compounds extraction from *S. thunbergii* were as follows: a liquid to solid ratio of 120 mL/g, an extraction time of 210 min, and an extraction temperature of 97 °C [41]. While Yuan et al. optimized the hot water extraction conditions for the extraction of bioactive compounds, in the present study, the UAE conditions for the extraction of bioactive compounds were optimized. Therefore, UAE conditions under short extraction time and low temperature were proven to be an effective extraction process for bioactive compounds compared to the previous hot-water extraction processes.

To verify the results, a confirmation experiment was conducted with three replicates at the optimum condition as predicted by the CCD model. When the experimental values of RSA, TIA, and CIA were evaluated under the optimal condition, they were 88.9% ± 3.11%, 85.1% ± 2.76%, and 89.7% ± 4.09%, respectively, and showed a strong agreement with the predictive model values (*p* > 0.05). Therefore, the experimental values were in good agreement with the predicted values, which proves the reliability of the UAE optimization results.

### 2.6. mRNA Expression of TRP-1, MMP-1, and MMP-9

TRP-1 functions as 5,6-dihydroxyindole-2-carboxylic acid oxidase, which is known to be the leading cause of skin pigmentation that acts by tyrosinase stimulation and eumelanin synthesis in epithelial cells [42]. In contrast, MMP-1 and MMP-9 break down type 1 collagen, which makes up 90% of the dermal layer, thereby causing collagen degradation, loss of elasticity, and skin aging [43].

**Figure 6:**
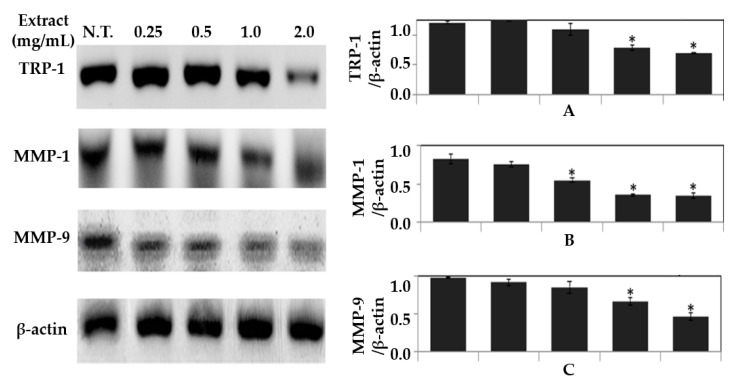
RT-PCR analysis for *TRP-1* (**A**), *MMP-1* (**B**), and *MMP-9* (**C**), and *β-actin* expression. B16-F0 cells were treated with various concentrations of *S. thunbergii* extract for 24 h. The statistical analysis of the data was carried out by use of a t-test (* *p* < 0.05).

In this study, *S. thunbergii* extract was produced using the optimal UAE condition established through a statistically based optimization, and the extract was tested on B16-F0 cell lines to evaluate skin-whitening and anti-wrinkling properties by comparing the mRNA expression levels of *TRP-1*, *MMP-1*, and *MMP-9*. The expression level of *TRP-1*, a major gene related to melanin synthesis, was found to be concentration-dependent in *S. thunbergii* extract and significantly decreased after treatment with 1 and 2 mg/mL of extract compared with the control group (*p* < 0.05) (Figure 6A).

We also found that the expressions of *MMP-1* and *MMP-9* decreased proportionally with the *S. thunbergii* extract concentration (Figure 6B,C). Particularly, the expression levels of *MMP-1* and *MMP-9* were inhibited by 58.6% and 78.8%, respectively, in the group treated with 2 mg/mL of *S. thunbergii* extract compared with the control groups (*p* < 0.05). From the above results, it was confirmed that *S. thunbergii* extract produced under optimal UAE conditions can effectively inhibit the mRNA expressions of *TRP-1*, *MMP-1*, and *MMP-9* in B16-F0 cell lines, thereby inhibiting melanin production and collagen decomposition.

### 2.7. Identification of Caffeic Acid in S. thunbergii Extract

**Figure 7:**
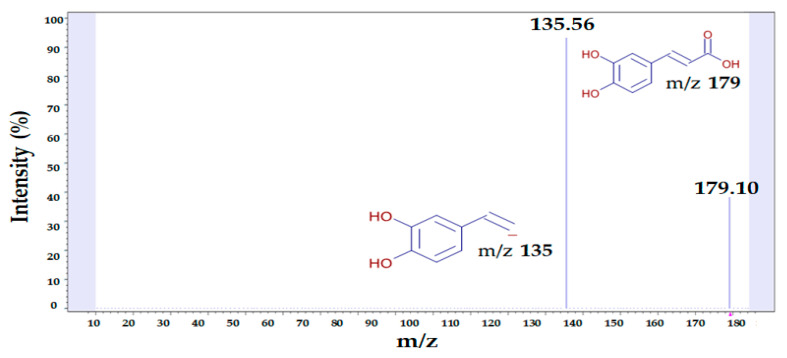
LC-MS/MS spectra of *S. thunbergii* extract and proposed fragmentation pattern of *m*/*z* 135.56 → 179.10 transitions (full ion scan in negative ion mode).

In a previous experiment, the UAE conditions to maximize the antioxidant, skin-whitening, and anti-wrinkling effects of *S. thunbergii* extract were optimized; however, further studies were needed to explore the bioactive ingredients in the extract. Therefore, phenolic compounds from *S. thunbergii* extract were identified using liquid chromatography–tandem mass spectrometry (LC-MS/MS), as this technology enables the accurate identification of phenolic compounds with structural characterization and the detection of small molecules in natural sources. The identification of the peaks was based on the retention time (RT), precursor ions, and related fragment ions of the standards. In the LC-MS/MS system, the caffeic acid showed a peak at 1.95 min of RT (Figure 7). 

In negative-ion mode, the *m*/*z* 179.10 ion, which showed one of the two ion peaks in the mass spectrum, corresponds to the molecular formula of caffeic acid and separated a fragment ion of *m*/*z* 135.56. Generally, after collision-induced dissociation, phenolic compounds produce a fragment ion characterized by the loss of CO_2_ (44 Da) from the carboxylic acid group. Due to this loss, subsequent cleavage of the 44-Da CO_2_ from the ion at *m*/*z* 179.10 gave the ion at *m*/*z* 135.56. Caffeic acid is a C6-C3 phenolic compound produced from phenylalanine or tyrosine by plants through the shikimate pathway of secondary metabolism and is a representative of the cinnamic acid (or phenylpropanoid) class. It enters the human diet through several vegetables and fruits [44]. In recent years, numerous studies have shown that the consumption of caffeic acid has numerous health benefits due to the antioxidant properties that help prevent various diseases associated with oxidative stress [45]. Thus, this study on phenolic compounds is very useful and may play an important role in the quality control process and future exploration of *S. thunbergii* as an ingredient with skin-whitening and anti-wrinkling properties.

## 3. Materials and Methods

### 3.1. Materials and Reagents

*S. thunbergii* collected from the south coast of Jeju Island, Korea, in October of 2019 was purchased in Para Jeju (Jeju, Korea). Prior to the experiment, *S. thunbergii* was powdered below 0.42 mm using a grinder (HMF-3000S, Hanil Co., Wonju, Korea) and stored in a refrigerator at −5 °C. Ethanol for solvent extraction was purchased from Samchun Chemical Co. (95.0 *v/v* %, Pyungtaek, Korea). Ascorbic acid (vitamin C), arbutin, and kojic acid used as standards for control tests were purchased from Sigma-Aldrich Co., Ltd. (St. Louis, MO, USA). All other chemicals used in this experiment were analytical grade.

### 3.2. UAE Process

**Figure 8:**
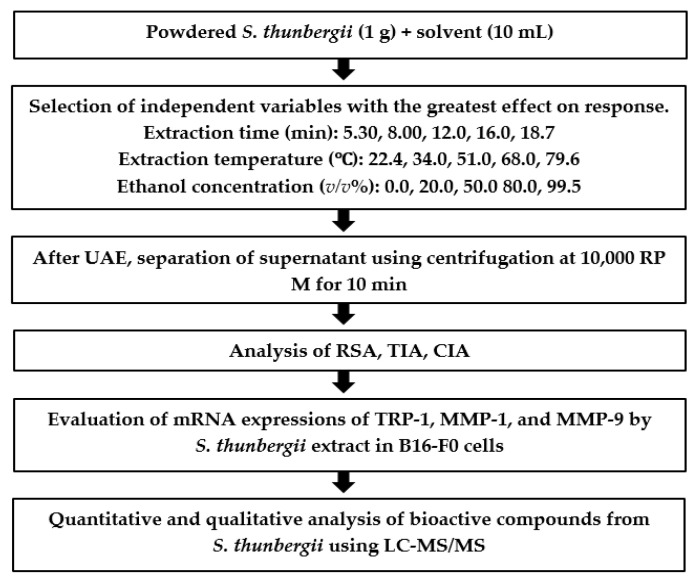
Flow chart showing the overall experimental design. RSM was used to optimize the levels of independent variables, that is, extraction time, extraction temperature, and ethanol concentration, on the UAE process.

Dried powder of the sample (1 g) was placed into a pressure vessel (XF100, Anton Paar Co., Ltd., Graz, Austria) with 10 mL of the solvent and mixed using a vortex mixer (VM-10, Daihan sci. Co., Wonju, Korea) for 1 min. These samples were extracted under 17 individual UAE conditions derived from CCD with an extraction time of 5.30~18.7 min, extraction temperature of 22.4~79.6 °C, and ethanol concentration of 0.0~99.5%. UAE was conducted using an ultrasound device (SD-D250H, Sungdong Co., Seoul, Korea) with an electric power of 200 W and a 40 kHz frequency equipped with a digital timer and a temperature controller. After extraction, the supernatant was separated at 10,000 rpm for 10 min using a centrifuge (1236R, Labogene Co., Daejeon, Korea). Then the solution was filtered through a cellulose acetate disk filter with porosity 0.45 µm and used for RSA, TIA, and CIA analyses (Figure 8). 

### 3.3. Experiment Design

Design-Expert software (Ver. 8.0, Stat-Ease, Minneapolis, MN, USA) was used to maximize the extraction of bioactive compounds from *S. thunbergii* through the optimization of UAE conditions using CCD. As independent variables, key variables including extraction time (X_1_), extraction temperature (X_2_), and ethanol concentration (X_3_) were selected, and they were coded into 5 (−1.68, −1, 0, 1, 1.68) levels, as shown in Table 1. RSA, TIA, and CIA were set as dependent variables affected by major independent variables. Experimental values were obtained under 17 conditions generated by the CCD, and the correlation of each independent and dependent variable was quantified using a quadratic regression equation [46]. The following quadratic regression equation was used to calculate dependent variable values according to changes in the independent variables:

(2) $$Y=\beta_{0}+\overset{k}{\underset{i=1}{\sum }}\beta_{i}X_{i}+\overset{k}{\underset{i=1}{\sum }}\beta_{ii}X_{i}^{2}+\overset{k}{\underset{i>1}{\sum }}\beta_{ij}X_{i}X_{j}$$

where *Y* represents the dependent variables (RSA, TIA, CIA), *β*_0_ is a constant coefficient, and k is a test variable. *β_i_*, *β_ii_*, and *β_ij_* are the regression coefficients for the linear, quadratic, and interaction terms, respectively.

To evaluate the predicted model on the independent variable, an analysis of variance (ANOVA) with a 95% confidence level was carried out to assess the effect of each variable including extraction temperature, time, and ethanol concentration. In addition, the regression coefficient (R^2^), the *p*-value of the regression model, was used to determine the fitness of the regression model [47].

### 3.4. Radical Scavenging Activity (RSA) Assay

The antioxidant effect of *S. thunbergii* extract was assessed based on their scavenging activity on 1,1-diphenyl-2-picrylhydrazyl (DPPH, Sigma-Aldrich) free radicals using a modified DPPH assay [48]. The stock solution was prepared by dissolving 0.1 M DPPH with methanol and then stored at room temperature. The diluted DPPH solution with methanol was prepared to obtain an absorbance of 1.0 ± 0.02 at 517 nm using a UV-vis spectrophotometer (Optizen 2120UV, Mecasys, Daejeon, Korea). A 1.25 mL aliquot of DPPH solution was mixed with 0.25 mL of diluted *S. thunbergii* extract (50–500 mg/mL) and allowed to stand at room temperature in the dark for 20 min. The change of absorbance was monitored at 517 nm, and the RSA was calculated using the following formula:

(3) $$\mathrm{RSA}\;\left(\%\right)=\left\{1-\frac{Abs\;\left(sample\right)}{Abs\;\left(control\right)}\right\}\times 100$$

where the *Abs*_(*control*)_ is the absorbance of the control and the *Abs*_(*sample*)_ is the absorbance of the extract. The same concentration of ascorbic acid (50–500 mg/mL) was used as a positive control.

### 3.5. Tyrosinase Inhibitory Activity (TIA) Assay

The TIA was measured according to the method reported by Yagi [49]. The reaction mixture contained 0.4 mL of sodium phosphate buffer (67 mM, pH 6.8), 0.2 mL of 10 mM 3,4-dihydroxy phenylalanine (L-DOPA, Sigma-Aldrich), 0.2 mL of mushroom tyrosinase (125 unit/mL, Sigma-Aldrich), and 0.2 mL of extract solution. The reaction was carried out at 25 °C for 30 min. After the reaction, absorbance was measured at 475 nm, and the results were compared with the control. The TIA was calculated according to the below equation:

(4) $$\mathrm{TIA}\;\left(\%\right)=\left\{1-\frac{Abs\;\left(sample\right)}{Abs\;\left(control\right)}\right\}\times 100$$

where the *Abs*_(*control*)_ is the absorbance of the control and the *Abs*_(*sample*)_ is the absorbance of the extract.

### 3.6. Collagemase Inhibitory Activity (CIA) Assay

The CIA assay was performed according to the method reported by Wünsch and Heindrich [50]. Collagenase (0.2 mg/mL, Sigma-Aldrich)was dissolved in 0.1 M Tris–HCl (pH 7.5). The substrate, 4-phenylazobenzyloxycarbonyl-Pro-Leu-Gly-Pro-Arg (0.4 mg/mL, Sigma-Aldrich), was dissolved in 0.1 M Tris–HCl (pH 7.5) containing 4 mM CaCl_2_. The reaction mixture for evaluating collagen hydrolysis contained collagenase (75 μL), sample (50 μL), and substrate solutions (125 μL). For the control group, 50 μL distilled water was added into the reaction mixture instead of the extract. The mixture was allowed to incubate at 37 °C for 30 min, and 0.25 mL of 25 mM citric acid was added for the termination of enzyme reactions. After mixing with ethyl acetate, the supernatant was separated, and the absorbance was measured at 320 nm. The percentage of inhibition was calculated according to the following formula:

(5) $$\mathrm{CIA}\;\left(\%\right)=\left\{1-\frac{Abs\;\left(sample\right)}{Abs\;\left(control\right)}\right\}\times 100$$

where the *Abs*_(*control*)_ is the absorbance of the control and the *Abs*_(*sample*)_ is the absorbance of the extract.

### 3.7. Validation of the Model

The optimized conditions for UAE (extraction time, extraction temperature, and ethanol concentration) were validated with the in vitro evaluation of the antioxidant activity, skin-whitening, and anti-wrinkle effects (RSA, TIA, and CIA) according to the values obtained from CCD. All the responses were again determined under the optimized condition of the UAE. The experimental values were compared with those predicted by the model in order to assess its validity. LC-MS/MS analysis was performed on the extracts generated under the optimal condition to find the main components in the *S. thunbergii* extract.

### 3.8. Cell Culture

B16-F0 melanoma cells were purchased from the Korean Cell Line Bank Co. (KCLB, Seoul, Korea) and were cultured in Dulbecco’s modified Eagle medium (DMEM, Gibco BRL Co., Ltd., Gaithersburg, MD, USA) content with 10% fetal bovine serum, and 1% penicillin (Thermo Fisher Sci. Inc., Waltham, MA, USA). Cells were incubated at 37 °C with 5% CO_2_ (MCO-5AC, Sanyo Co., Ltd., Tokyo, Japan) and grown as a monolayer in 25 cm^2^ culture flasks. When a cell line reached about 80% confluence, subculturing was performed by treating with trypsin-EDTA to obtain single cells to ensure proper growth and health of the cells.

### 3.9. Reverse Transcription Polymerase Chain Reaction (RT-PCR)

For performing RT-PCR, 1.0 × 10^6^ cells were plated per well of a 24-well plate. Total RNA was extracted from cells with an AccuPrep universal RNA extraction kit (Bioneer Co., Daejeon, Korea). Reverse transcription was performed with 0.5 μg of total RNA for cDNA synthesis using the amfiRivert cDNA synthesis platinum master mix (GenDEPOT Co., TX, USA). cDNA was amplified with each primer, such as *TRP-1*, *MMP-1*, *MMP-9*, and *β-actin* (Table 5). PCR was performed in a 20 µL volume containing 1 μL cDNA, 10 μL Taq Premix (Genet bio, Daejeon, Korea), and 9 μL diethylpyrocarbonate (DEPC). The PCR conditions were as follows: 94 °C for 5 min, followed by 25 cycles at 95 °C for 5 s, 60 °C for 31 s (for *TRP-1*) or 55 °C for 30 s (for *MMP-1*) or 59 °C for 30 s (for *MMP-9*), and 72 °C for 30 s extension. Each PCR product was electrophoresed on 1% agarose gel and visualized by using the Gel Doc TM XR+system and quantity one software (Bio-Rad Co., Hercules, CA, USA). The *β-actin* as a housekeeping gene was used to normalize the expression levels of *TRP-1*, *MMP-1*, and *MMP-9*.

### 3.10. LC-MS/MS Analysis

The chromatographic separation of *S. thunbergii* extract was performed using a Finnigan Surveyor Plus HPLC System (Thermo Electron Corporation, San Jose). Separation was achieved by using a ROC C18 column with 150 mm column length, 3 mm internal diameter and 3 μm particle size (RESTEK Co., Bellefonte, PA, USA) while using a gradient elution of 0.1% formic acid in water (mobile phase A) and 0.1% formic acid in acetonitrile (mobile phase B) at a flow rate of 0.2 mL/min, as follows: 5% to 100% mobile phase B for 11 min, 100% to 5% mobile phase B for 4 min, 37% mobile phase B for 2 min, 37% to 10% mobile phase B for 0.1 min, and 10% mobile phase B for 2.4 min. The injection volume was 10 μL, and the column was maintained at 30 °C. Mass spectrometric experiments were performed using a Thermo Finnigan TSQ Quantum Ultra EMR triple quadrupole mass spectrometer (Thermo Fisher Sci. Inc., Waltham, MA, USA). The *S. thunbergii* extract was analyzed by negative ion electrospray ionization using electrospray ionization (ESI), specifically utilizing the turbo ion spray mode. The ESI source settings for the ionization of the *S. thunbergii* extraction in the negative mode were as follows: gas temperature, 270 °C; gas flow, 19 L/min; sheath gas temperature, 400 °C; sheath gas flow, 10 L/min; capillary voltage, 3000 V; nozzle voltage, 1000 V. Mass spectra were recorded in the negative ion mode between 100 and 500 *m*/*z* using nitrogen as the collision gas. The analysis of main components in *S. thunbergii* extract was conducted by comparing the obtained molecular ions and fragmentation patterns of LC-MS/MS result with data from the literature and with a mass library for the standard compounds.

## 4. Conclusions

This study proposed optimal conditions for the UAE process that can maximize antioxidant, skin-whitening, and anti-wrinkling effects for the production of value-added bioactive compounds from *S. thunbergii*, which are widespread in the subtropical coast of Southeast Asia, causing marine pollution and ecological disturbance. The most influential variable in performing UAE optimization was ethanol concentration, which confirmed that the use and concentration determination of binary solvents consisting of water and ethanol were an important consideration in the UAE. When overlapping each response surface for the simultaneous optimization of RSA, TIA, and CIA, an extraction time of 12.0 min, an extraction temperature of 65.2 °C, and an ethanol concentration of 53.5% were predicted, under which conditions RSA values of 86.5%, TIA values of 88.3%, and CIA values of 91.4% were identified.

When the effects of *TRP-1*, *MMP-1*, and *MMP-9* on expression were evaluated at the mRNA level using *S. thunbergii* extract produced under optimal UAE conditions, it was confirmed that *S. thunbergii* extract can decrease mRNA levels of *TRP-1*, *MMP-1*, and *MMP-9* and thereby prevent melanin production as well as skin collagen decomposition.

Thus, *S. thunbergii* extract is expected to be widely utilized as a new source from marine biomass in the production of functional ingredients for cosmetics, food, and medicines. Additionally, the process of extracting bioactive compounds using UAE is believed to provide fundamental data on the process development and contribute to the determination of optimal extraction conditions in the production of new functional ingredients from *S. thunbergii* and other macroalgae.

## Figures and Tables

**Table 1:** Independent variables and coded values used for the optimization of the UAE condition of *S. thunbergii*.

X_i_	Independent Variables	Coded and Experimental Levels				
		−1.68	−1	0	+1	+1.68
X_1_	Extraction time (min)	5.30	8.00	12.0	16.0	18.7
X_2_	Extraction temperature (°C)	22.4	34.0	51.0	68.0	79.6
X_3_	Ethanol concentration (*v/v* %)	0.0	20.0	50.0	80.0	99.5

The level of each variable was selected based on our preliminary experiments by a one-factor-at-a-time method. The distance of the axial points from the center point was ±1.68.

**Table 2:** Independent variables and their responses (experimental data) obtained from 17 experimental combinations of CCD.

Run No.	Extraction Conditions			RSA (%)	TIA (%)	CIA (%)
	X_1_	X_2_	X_3_			
1	8.00	34.0	20.0	65.2	71.2	58.7
2	16.0	34.0	20.0	42.1	66.1	48.3
3	8.00	68.0	20.0	52.7	67.1	83.4
4	16.0	68.0	20.0	67.7	72.4	69.9
5	8.00	34.0	80.0	10.3	75.7	83.7
6	16.0	34.0	80.0	8.90	78.4	80.8
7	8.00	68.0	80.0	21.0	84.7	80.3
8	16.0	68.0	80.0	17.6	80.5	92.3
9	5.30	51.0	50.0	66.5	85.4	79.3
10	18.7	51.0	50.0	88.2	83.9	71.8
11	12.0	22.4	50.0	37.7	74.9	62.4
12	12.0	79.6	50.0	82.1	92.6	84.8
13	12.0	51.0	0.0	87.8	55.3	48.1
14	12.0	51.0	99.5	2.37	86.1	78.2
15	12.0	51.0	50.0	88.7	89.8	88.9
16	12.0	51.0	50.0	89.9	83.6	83.7
17	12.0	51.0	50.0	88.3	86.6	89.9

X_1_: extraction time (min), X_2_: extraction temperature (°C), X_3_: ethanol concentration (%).

**Table 3:** Quadratic regression equations calculated by CCD for the optimization of UAE conditions.

Responses	Quadratic Regression Equations	R^2^	*p* Value
RSA (%)	Y _(RSA)_ = 90.65 + 1.72X_1_ + 7.85X_2_ − 23.26X_3_ + 4.51X_1_X_2_ + 0.41X_1_X_3_ + 0.79X_2_X_3_ − 9.20X_1_^2^ − 15.37X_2_^2^ − 21.40X_3_^2^	0.8554	0.0283
TIA (%)	Y _(TIA)_ = 7.06 − 0.28X_1_ + 3.16X_2_ + 6.88X_3_ + 0.43X_1_X_2_ − 0.21X_1_X_3_ + 1.11X_2_X_3_ − 1.75X_1_^2^ − 2.07X_2_^2^ − 6.92X_3_^2^	0.8591	0.0262
CIA (%)	Y _(CIA)_ = 88.00 − 1.42X_1_ + 7.33X_2_ + 9.92X_3_ + 2.47X_1_X_2_ + 5.10X_1_X_3_ − 3.78X_2_X_3_ − 3.17X_1_^2^ + 3.87X_2_^2^ + 7.71X_3_^2^	0.9237	0.0037

A negative coefficient in each quadratic regression equation represents an antagonistic effect of the variables, and a positive coefficient represents a synergistic effect of the variables. X_1_: extraction time (min), X_2_: extraction temperature (°C), X_3_: ethanol concentration (%).

**Table 4:** ANOVA for the quadratic regression equations to test the significance and adequacy of the models on RSA, TIA, and CIA.

Variables	RSA (%)			TIA (%)			CIA (%)		
	Sum of Squares	F	*p*	Sum of Squares	F	*p*	Sum of Squares	F	*p*
Model	14,271.9	4.60	0.0283	1317.41	4.74	0.0262	3161.66	9.42	0.0037
X_1_	40.49	0.12	0.7418	1.10	0.036	0.8556	27.35	0.73	0.4201
X_2_	840.99	2.44	0.1622	136.40	4.42	0.0736	734.15	19.69	0.0030
X_3_	7300.46	21.19	0.0025	639.25	20.71	0.0026	1329.55	35.65	0.0006
X_1_X_2_	162.76	0.47	0.5140	1.49	0.048	0.8323	48.94	1.31	0.2896
X_1_X_3_	1.35	3.92 × 10^−3^	0.9518	0.34	0.011	0.9192	208.37	5.59	0.0501
X_2_X_3_	5.03	0.015	0.9072	9.81	0.32	0.5905	114.53	3.07	0.1232
X_1_^2^	957.11	2.78	0.1395	34.67	1.12	0.3244	113.83	3.05	0.1241
X_2_^2^	2672.79	7.76	0.0271	48.44	1.87	0.2506	169.84	4.55	0.0703
X_3_^2^	4942.54	14.34	0.0068	516.08	16.75	0.0046	641.23	17.19	0.0043

*p*-value < 0.05 is significant at α = 0.05. X_1_: extraction time (min), X_2_: extraction temperature (°C), X_3_: ethanol concentration (%).

**Table 5:** List of primers used to determine gene expressions of *TRP-1*, *MMP-1*, and *MMP-9* using RT-PCR. The sequence of designed primers for each gene is shown as forward and reverse.

Primer	Forward (5′-3′)	Reverse (5′-3′)	Size (bp)
*TRP-1*	GCTGCAGGAGCCTTCTTTCTC	AAGACGCTGCACTGCTGGTCT	268
*MMP-1*	AACTTTGACACCGTGGCCA	CAATGGGCATTGGGTACC	108
*MMP-9*	AGTTTGGTGTCGCGGAGCAC	TACATGAGCGCTTCCGGCAC	754
*β-actin*	AGCACAGAGCCTCGCCTTT	CTTAATGTCACGCACGATTTCC	697

## Data Availability

Not applicable.
